# An image‐based mapping of significance and relevance of facial skin colour changes of females living in Thailand

**DOI:** 10.1111/ics.12593

**Published:** 2019-12-25

**Authors:** P. Séroul, R. Campiche, S. Gougeon, M. Cherel, A.V. Rawlings, R. Voegeli

**Affiliations:** ^1^ Newtone Technologies 13 bis place Jules Ferry Lyon F‐69006 France; ^2^ DSM Nutritional Products, Personal Care & Aroma Wurmisweg 576 Kaiseraugst CH‐4303 Switzerland; ^3^ AVR Consulting Ltd 26 Shavington Way Kingsmead Northwich Cheshire CW98FH UK

**Keywords:** claim substantiation, facial mapping, relevance, significance, skin colour, statistics

## Abstract

**Objective:**

There are methods to evaluate skin colour on defined areas over the face but no approach automatically and accurately evaluates skin colour variations on large facial areas, comparing subjects, treatments and/or time points. We propose such an image‐based approach to visualize quickly the outcome of clinical studies on colour variations.

**Methods:**

Among 54 Asian women, one group applied a vehicle twice daily, during 28 days, and the other group an anti‐ageing emulsion, taking facial images at baseline and after treatment. Changes in L*a*b* values were studied on four pre‐selected facial regions. We also reconstructed average facial images from which the L*a*b* parameters were extracted for every pixel, computing relevance (Δ*E*) and significance data. Using colour gradients, we mapped these results onto the average facial images.

**Results:**

After treatment, L*a*b* parameters show no statistically relevant colour changes in the vehicle group. In the ‘active’ group, skin was lighter at the upper cheek and, overall, redness decreased. Relevance and significance maps confirmed no visible colour changes in the vehicle group. In the ‘active’ group, the mapping approach revealed colour changes and their location. Skin became lighter below the eye, cheek and forehead. It was less red below the eyes, on the cheek, jawline and forehead, and generally more yellow.

**Conclusion:**

Our image‐based mapping approach proves to be powerful. It enables us to identify precise facial regions of relevant and statistically significant colour changes after a topical treatment, regions that would have otherwise been undetected.

## Graphical abstract

We recently introduced facial colour mapping to visualize differences in skin parameters across the face. Here, we introduce facial colour mapping that enables the visualization of relevance and statistical significance of such differences.

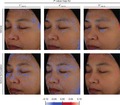

## Résumé

**Objectif:**

Il existe des méthodes pour évaluer la couleur de la peau sur des zones pré‐définies du visage mais aucune approche n'évalue de manière automatique et précise les variations de couleur de peaux sur de large régions du visage, en comparant les sujets, les traitements et/ou les temps d'analyse. Nous proposons une telle méthode basée sur l'analyse d'images pour visualiser de manière rapide les résultats des études cliniques portant sur des variations colorimétriques.

**Méthodes:**

Parmi 54 femmes d'origine asiatique, un premier groupe a appliqué un véhicule deux fois par jour, pendant 28 jours. Un deuxième groupe a, lui, appliqué une émulsion anti‐âge. Des images de visage ont été réalisées avant et après traitement. Les variations des valeurs L*a*b* ont été étudiées sur quatre régions du visage pré‐sélectionnées. Nous avons également reconstruit des images de visages moyens pour lesquelles les paramètres L*a*b* ont été extraits pour chaque pixel. Pour ces mêmes pixels, les valeurs de pertinence (delta E) et significativité ont été calculées. A l'aide d'un gradient de couleur, nous avons représenté ces résultats sur les images de visages moyens.

**Résultats:**

Après traitement, les paramètres L*a*b* n'ont montré aucun résultat significativement pertinent pour le groupe ayant appliqué le véhicule. Pour le groupe "actif", la peau est devenue plus claire sur la partie supérieure de la joue et globalement moins rouge. Les cartographies de pertinence et de significativité ont confirmée l'absence de variation colorimétrique sur le groupe véhicule. Sur le groupe "actif", l'approche par cartographie a révélé les changements de couleurs et leur localisation. La peau est devenue plus claire sous les yeux, sur les joue et le front. Le dessous des yeux, les joues, la mâchoire et le front sont devenues moins rouges et généralement plus jaunes.

**Conclusion:**

Notre approche de cartographie basé sur l'analyse d'images s'est montrée pertinente. Elle nous permet d'identifier de manière précise les régions du visage sur lesquelles des changements pertinents et significatifs de couleur ont eu lieu après l'application d'un traitement topique. Ces régions n'auraient pas été détectées sans cette technique.

## Introduction

Skin appearance and colour result from the perception of light reflected by interactions at and beneath the skin surface. Reflection and scattering by the stratum corneum are caused by the optical heterogeneity of the skin surface, providing information about its roughness, scaliness and microstructure but also about its hydration state and sebum level 1, 2. Part of the light reaches deeper layers of the skin, epidermis and dermis, where it undergoes sub‐surface scattering and absorption by skin chromophores: mostly melanin, but also haemoglobin, oxy‐haemoglobin, bilirubin and carotenoids 1, 3, 4. Chromophore concentration can be altered by several endogenous or exogenous factors (UV light, drugs, irritants…) and, with age, skin conditions deteriorate as degenerative events occur, leading to visible signs that increase perceived age 5, 6. Therefore, examination of skin colour is a key parameter for dermato‐cosmetic research and provides important insights into skin physiology, disorders or pathologies 7, 8, 9, 10. Moreover, it enables monitoring of the effects of skin care treatments 11, 12.

Colour perception is a subjective, non‐linear, sensory perception that varies between individuals 13, 14. Therefore, to objectively determine skin colour and circumvent the limitations of evaluation by trained experts, different instrumental approaches have been developed. One approach relies on the direct analysis of the light remitted by a region of interest upon illumination. Among the devices using this approach, some only measure a few wavelength bands, providing approximate quantification of chromophores 11, 15, 16, while others analyse the complete light spectrum 11, 17, 18, 19. Still, except for the most recent devices equipped with a charge coupled device (CCD) camera, the result is the average value of the entire region studied, in general a circular surface of about 0.5 cm^2^. Nevertheless, skin is not homogeneous: hair, blood vessels, hyper‐ or hypo‐pigmented spots can lead to significant site‐related variations. Another approach uses image analysis that relies on the acquisition of digital colour images according to three broad band filters – red, green and blue – that mimics light sensing by the human eye 20, 21. It offers the advantage of studying large skin areas without any contact and can provide quantitative data for every pixel of an image 22, 23. Despite the many applications of the devices using the imaging approach, limitations exist. This is the case to faithfully detect local variations in colour changes among populations, extended periods of time and/or to compare the effect of treatments. To the best of our knowledge, there is no approach enabling reproducible visualization and detection of these variations or a reliable statistical analysis of these variations.

We recently established a unique procedure that continuously map on facial images data from skin parameters such as skin hydration, transepidermal water loss and skin surface pH 24, 25. This approach enables direct visualization of local variations in the various parameters, therefore, proving to be a powerful tool to better describe visualize and compare complex data. Inspired by this technology, we developed and tested an image‐based approach that, from high quality reconstructed average faces obtained from digital pictures of several subjects taken at different time points (*e.g.* before and after a skin care treatment), automatically computes the L*a*b* parameters of every pixel for each time point, matching these parameters to the very pixels between subjects and time points. It then continuously maps skin colour changes over time as colour gradients, indicating the relevance of colour changes (intensity of colour changes between time points expressed as Δ*E*‐values) or significance of colour changes (statistical significance of colour changes between time points expressed as p‐values). We tested this new approach in a vehicle‐controlled 28‐day clinical study using a cream with a known anti‐ageing peptide.

## Materials and methods

### Subjects and treatment

Fifty‐four Asian women from Bangkok were recruited. The criteria for inclusion were an age ranging between 41 and 55 years old (47.4 ± 3.6 years old), no visible facial skin lesions and an average ITA° over the whole face of 0.7 ± 12 26. The study took place between 22 September and 22 October 2015 and the Guidelines of the Declaration of Helsinki were applied: all subjects were informed of the purpose of the study, received detailed information about the procedures and gave written informed consent prior to enrolment.

The study was a blinded, vehicle‐controlled, full face, parallel‐grouped trial. In the 28‐day application phase, the subjects applied either the vehicle or the test emulsion containing an anti‐ageing peptide with claimed effects on wrinkles and fine lines 27 (Table 1) twice daily, once in the morning and once in the evening, under normal conditions of use.

**Table 1:** Composition of the vehicle and of the active formulation emulsions

INCI Name	Vehicle (% w/w)	Active (% w/w)
Potassium cetyl phosphate	1.00	1.00
Cetyl alcohol	3.00	3.00
Cetyl palmitate	1.50	1.50
Octyldodecanol	3.00	3.00
Acrylates/C10‐30 alkyl acrylate crosspolymer	0.10	0.10
Butylene glycol	3.00	3.00
Aqua	84.81	80.81
Cyclopentasiloxane, Cyclohexasiloxane	2.50	2.50
Aqua, Sodium hydroxide	0.09	0.09
Phenoxyethanol, Ethylhexylglycerin	1.00	1.00
Perfume	0.05	0.05
Dipeptide diaminobutyroyl benzylamide diacetate, glycerine, aqua	0	4.00

### Acquisition of facial images and pre‐processing

At baseline and after the 28‐days treatment, homogeneous cross‐polarized pictures of both facial sides with a 30° angle were taken using a Visia‐CR^®^ device (Canfield Scientific, NJ, USA) and saved as high‐resolution (3648 × 5472px) jpeg files. The position of the face was stabilized by holders. All pictures included a 48‐colours reference chart (ColorChart, Newtone Technologies, Lyon, France).

To prevent that small variations in lighting affecting colour analysis, a colour consistency analysis was performed using the colour chart. The differences between the known colour reference and the colour reference data from the pictures were used to perform colour corrections using the ColorSkin software (Newtone Technologies, Lyon, France).

### Classical determination of L*, a* and b* colour parameters

**Figure 1:**
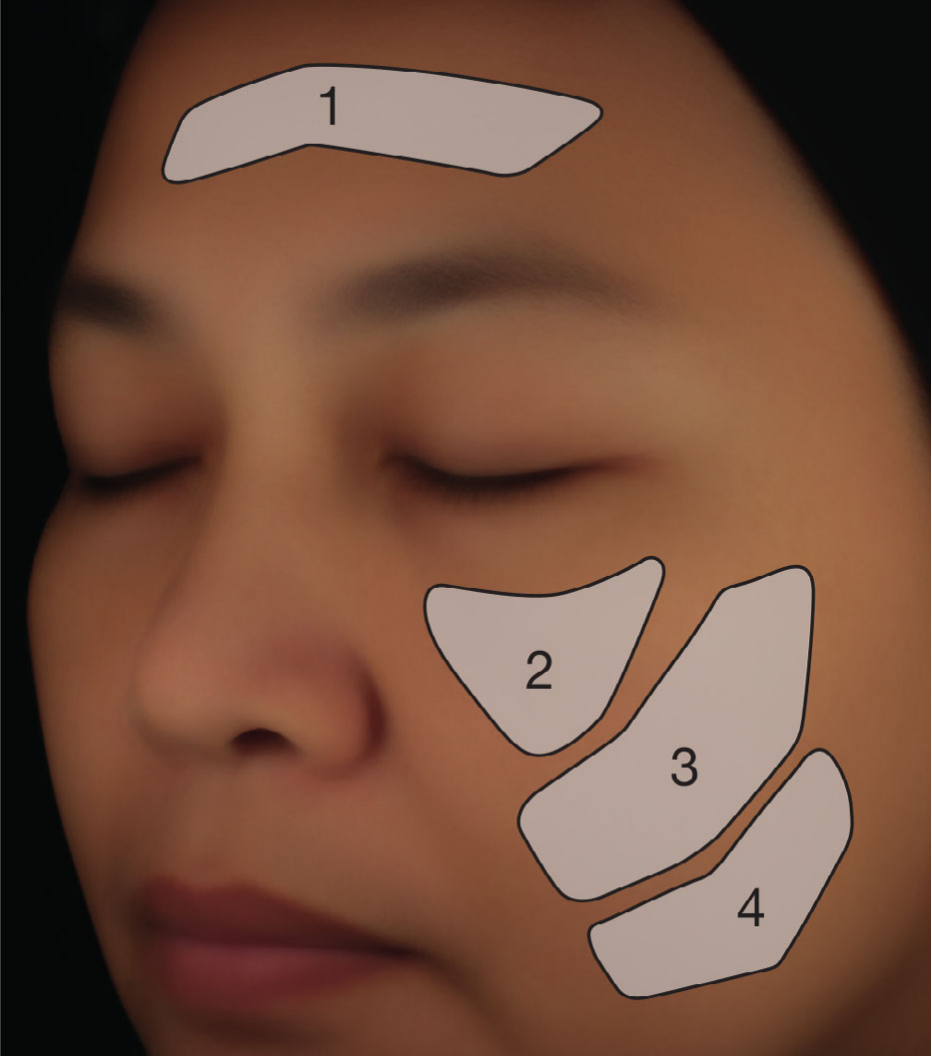
Localization of the four predefined regions of interest used for the determination of L*, a* and b* colour parameters according to the classical method.

Initial analysis of colour changes over the two time points focuses on four predefined regions of interest (Fig. 1): the forehead, the upper cheek, the middle cheek and the lower cheek. These regions were delimited on individual left and right face pictures of each subject using a template matching algorithm (Newtone Technologies, Lyon, France), and the average L*, a* and b* parameters over each entire region of interest was determined. These data were used to calculate the variations in the L*, a* and b* parameters between both time points for each subject. Finally, significance of colour changes was calculated by performing a paired Student’s *t*‐test or a Wilcoxon signed‐rank test on these differences, differentiating the vehicle‐treated group and the group treated with the emulsion containing the anti‐ageing peptide. *P*‐values below 0.05 were considered significant.

### Image‐based mapping of relevance and significance of changes in skin colour

**Figure 2:**
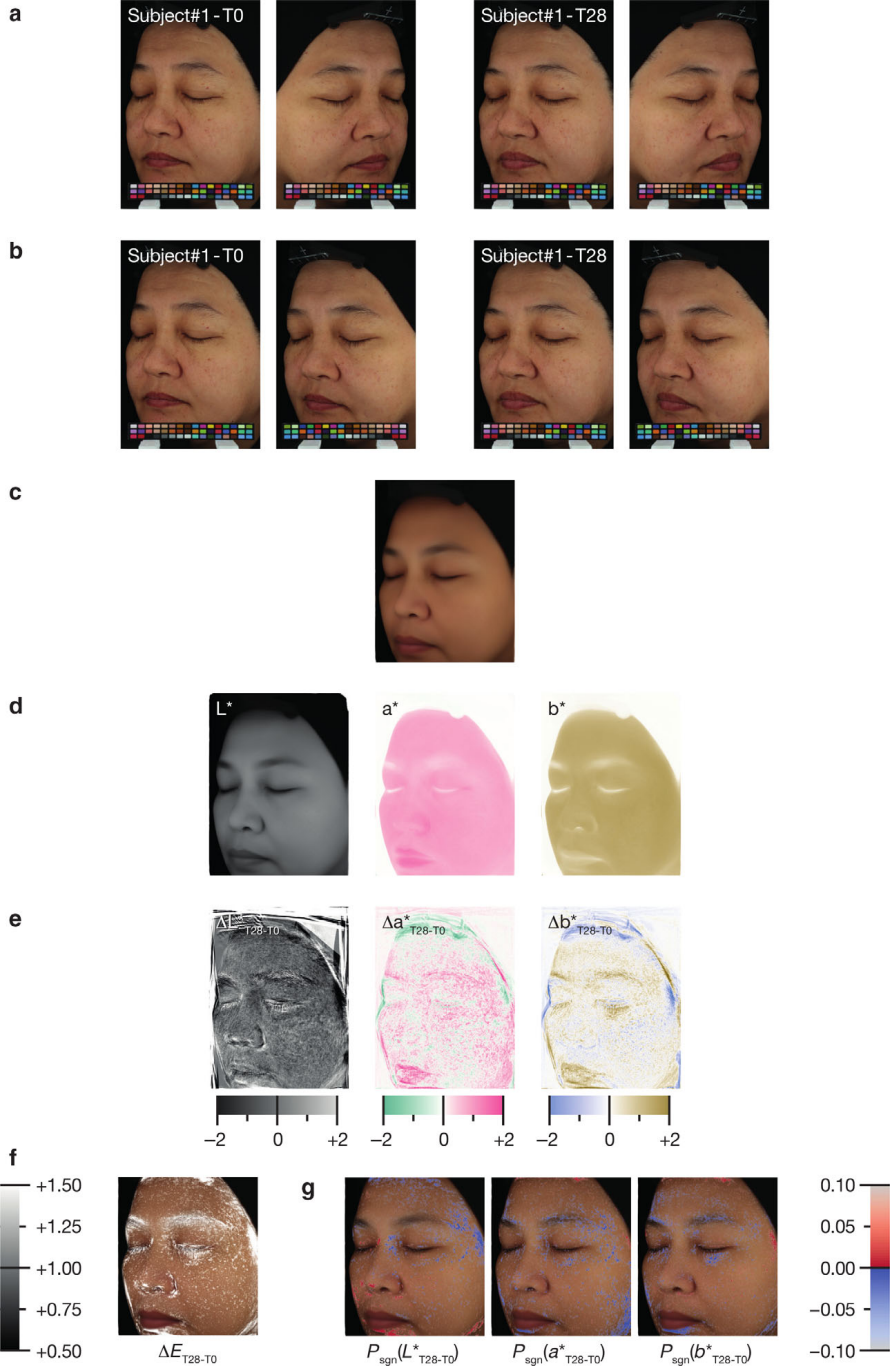
Illustration of the main steps involved in the computation of relevance map and significance map of colour changes. (a) Individual pictures of subjects taken at two time points (day 0 and day 28). (b) Individual pictures of subjects taken at the two time points with the mirror image of the right facial side used for computation of the average facial side image. (c) Average facial side reconstructed with spatial registration of characteristic morphological points. (d) Calculation of the L*, a* and b* colour parameters from the reconstructed average facial side images. (e) Computation of the variation in colour parameters between both time points ($\Delta L_{\text{T28}\;-\;\text{T0}}^{*}$, $\Delta a_{\text{T28}\;-\;\text{T0}}^{*}$ and $\Delta b_{\text{T28}\;-\;\text{T0}}^{*}$). (f) Relevance map: mapping, according to a greyscale, of Δ*E* results over the value of 1 on the reconstructed average facial side images. (g) Significance maps: mapping, according to a colour scale, of significant results (*P* < 0.05) from the paired Student’s *t*‐test on L*, a* or b* changes on the reconstructed average facial side images.

The first step in the image‐based mapping approach we developed was the reconstruction of a high‐resolution average facial side image. For this, we used the pictures of both facial sides from subjects of both groups and both time points (Fig. 2a). We reconstructed the average facial side image using the left facial side images and a horizontal symmetry of the right facial side images (Fig. 2b).

**Figure 3:**
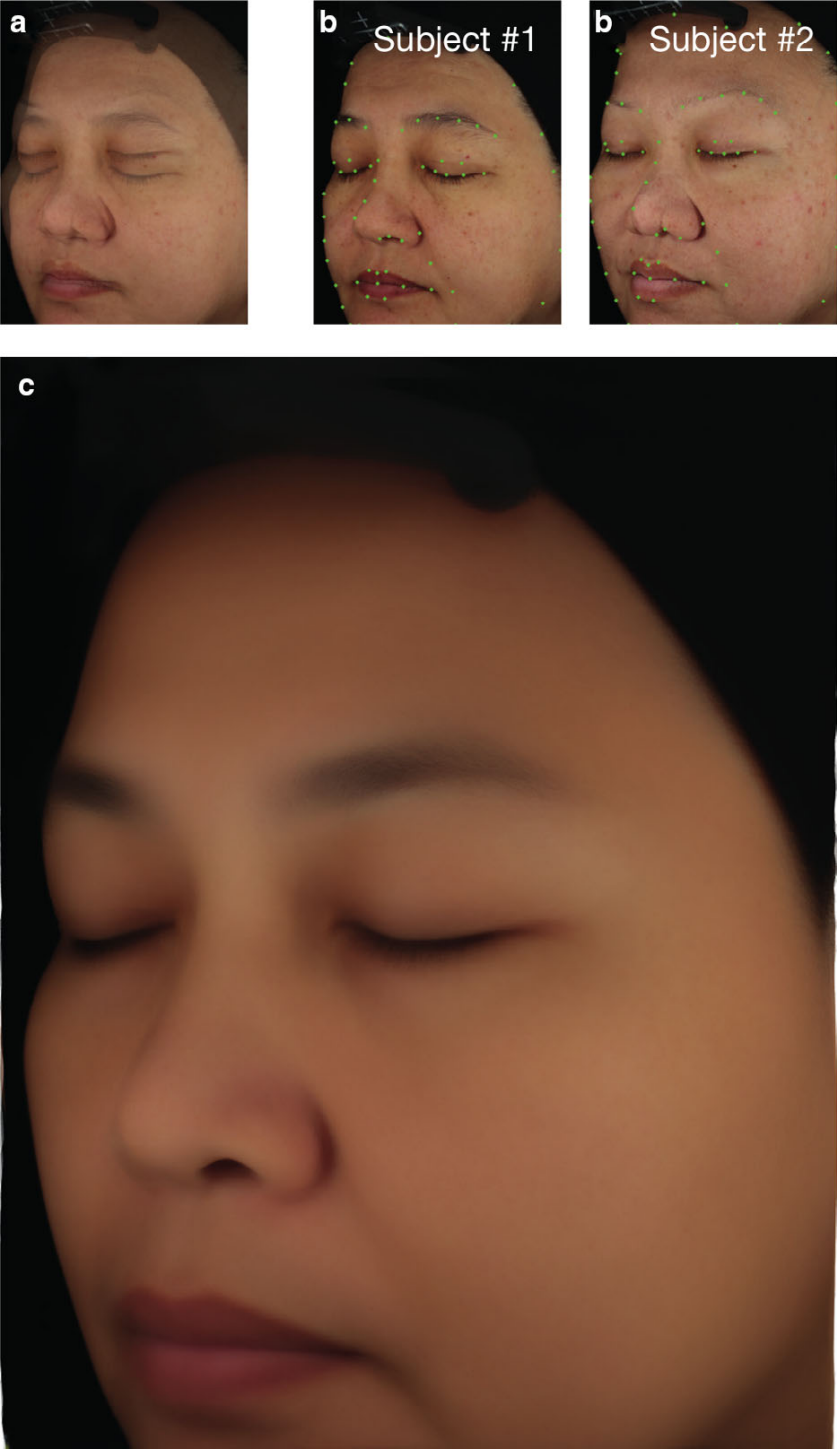
Illustration of the main steps involved in the computation of the high‐resolution average facial side image. (a) From left to right, average face reconstruction without spatial registration of characteristic morphological points in the case of two subjects. (b) Automatic detection of characteristic morphological points on individual faces. (c) High‐resolution average face reconstructed with spatial registration of characteristic morphological points (case of all subjects at T0).

The average facial side reconstruction process relies on the automatic detection of 60 characteristics morphological points on every individual image 28, 29. Characteristic morphological points of every image are then spatially registered in a common reference space using a diffeomorphic deformation algorithm 30, 31, 32. Finally, a statistical analysis performed on every single pixel ensures the best spatial and colorimetric consistency resulting in a high‐resolution average facial side image (Figs. 2c and 3) 33. In a last step, we used this global high quality reconstructed facial side image to extract a reconstructed average facial side image for each treatment and each time point.

We extracted the mean CIE L*, a* and b* colour parameters of every pixel from each reconstructed average facial side image (Fig. 2d) and calculated for every pixel the standard deviation over the subjects of each group. The means were then used to compute, for every pixel, the variations between the value of L*, a* and b* parameters between both time points ($\Delta L_{\text{T28}\;-\;\text{T0}}^{*}$, $\Delta a_{\text{T28}\;-\;\text{T0}}^{*}$ and $\Delta b_{\text{T28}\;-\;\text{T0}}^{*}$, Fig. 2e).

From the variations in the L*, a* and b* parameters over both time points, we calculated a relevance map of colour changes for both subject groups. For this, we calculated the Δ*E*‐values of every single pixel and converted the results, according to a greyscale. We finally produced the relevant colour change maps by applying on the reconstructed average facial side images only the grey scale results corresponding to Δ*E*‐values over a value of 1. This value is considered as a threshold for visible colour change for expert graders (Fig. 2f) 34.

Similarly, we used the variations in the L*, a* and b* parameters over both time points to generate significance maps of changes for each of these three parameters. For every single pixel of the reconstructed average facial side images and for each of the L*, a* and b* parameters, we calculated the *P*‐value from a paired Student’s *t*‐test on the differences over both time points: *P*_sgn_($L_{\text{T28}\;-\;\text{T0}}^{*}$), P_sgn_($a_{\text{T28}\;-\;\text{T0}}^{*}$) and P_sgn_($b_{\text{T28}\;-\;\text{T0}}^{*}$) and converted the results onto a blue‐red colour gradient scale. Finally, to represent the significance of the changes of each L*, a* and b* parameters, we only mapped onto the reconstructed average facial side images the blue‐red values corresponding to significant (*P* < 0.05) colour changes (Fig. 2g).

For the blue‐red colour gradient scale of significance maps, we arbitrarily set the blue gradient colour to the desired effect of an anti‐ageing compound, and the red to the opposite effect. Therefore, for the significance map of the L* parameter, we set positive *P*‐values, in blue, to significantly increased L* values, meaning significantly a lighter skin colour, and negative *P*‐values, in red, to significantly lower L* values, namely a significantly darker skin colour. For the significance map of the a* parameter, we set positive *P*‐values, in blue, to significantly decreased a* values, meaning a significantly less red skin colour, and negative *P*‐values, in red, to significantly higher a* values, namely a significantly redder skin colour. Finally, for the significance map of the b* parameter, we set positive *P*‐values, in blue, to significantly decreased b* values, meaning a significantly less yellow skin colour, and negative *P*‐values, in red, to significantly higher b* values, namely a significantly more yellow skin colour. Using this strategy, the resulting significance maps not only provide information on the significance of the changes over time, but also on the direction of colour changes over time.

## Results

### Classical L*a*b* colour analysis only shows changes for subjects treated with the active formulation

As a first step, we analysed changes in L*, a* and b* colour parameters over time using a classical method that determines the average value of these parameters over an entire region of interest. For this, we focused on 4 predefined regions (Fig. 1): the forehead (i), the upper cheek (ii), the middle cheek (iii) and the lower cheek (iv).

Results (Table 2) show that subjects who applied the vehicle for 28 days presented no significant colour changes in any of the facial regions studied. Only a trend for a slightly decreased yellowness could be detected on the forehead (Δb* = −0.24 ± 0.73, *P* = 0.051). On the contrary, subjects who applied the active formulation present several significant colour changes after 28 days. The most striking one is a decreased a* value over all regions studied, even if this decreased redness is only a trend (*P* = 0.057) at the level of the cheek. They also show lighter skin at the level of the upper cheek (ΔL*=0.36 ± 1.05, *P* = 0.016), and a trend for such a lightening at the level of the forehead (*P* = 0.064). Finally, the forehead shows increased yellowing (Δb* = 0.25 ± 0.83, *P* = 0.014).

**Table 2:** Evolution and significance of the evolution in the average L*, a* and b* colour parameters in the four pre‐defined regions of interest (Fig. 1) between day 0 and day 28. *P*‐values < 0.05 (in bold) are considered significant

Facial area	Treatment	ΔL*	*P*‐value	Δa*	*P*‐value	Δb*	*P*‐value
Forehead	Vehicle	−0.05 ± 1.05	0.705	−0.06 ± 0.93	0.443	−0.24 ± 0.73	0.051
	Active	+0.36 ± 1.38	0.064	−0.24 ± 0.85	**0.040**	+0.25 ± 0.83	**0.014**
Upper cheek	Vehicle	−0.13 ± 1.36	0.744	+0.09 ± 1.17	0.082	−0.06 ± 1.07	0.702
	Active	+0.36 ± 1.05	**0.016**	−0.35 ± 0.93	**0.018**	+0.13 ± 1.25	0.318
Middle cheek	Vehicle	+0.10 ± 1.33	0.185	−0.12 ± 1.04	0.071	+0.07 ± 0.98	0.615
	Active	+0.29 ± 1.21	0.081	−0.26 ± 0.94	0.057	+0.22 ± 1.04	0.206
Lower cheek	Vehicle	+0.07 ± 1.42	0.502	−0.13 ± 1.03	0.360	−0.03 ± 0.97	0.884
	Active	+0.09 ± 1.45	0.671	−0.29 ± 0.91	**0.026**	+0.17 ± 1.02	0.084

### Relevance maps indicate visible colour changes but gives little information on the nature of the changes

**Figure 4:**
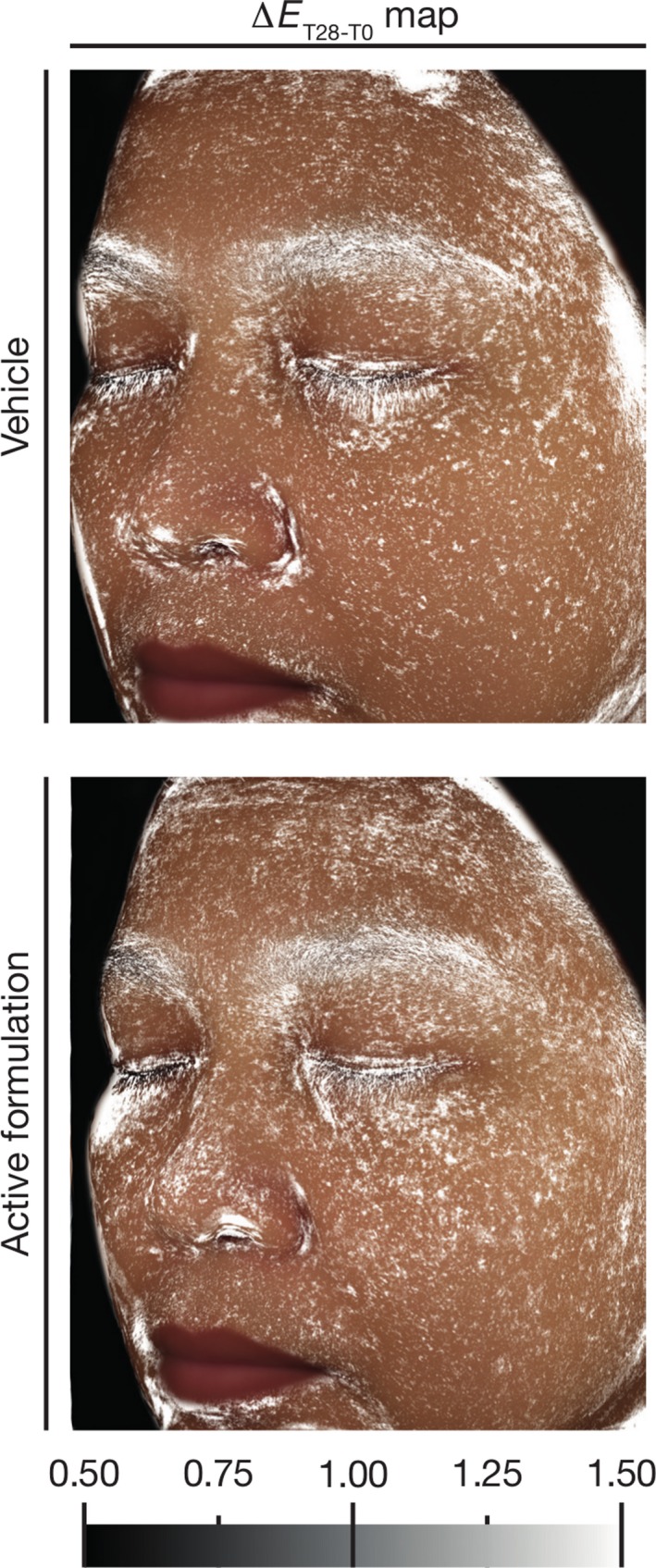
Relevance map of colour changes (Δ*E* > 1) in the group which tested the vehicle emulsion and in the group which tested the active formulation.

We then used our image‐based approach to map the relevance of skin colour changes over the two time points, calculating the Δ*E* of every pixel from a reconstructed average facial side image and mapping, according to a greyscale, Δ*E*‐values over 1. These values correspond to visible colour changes (Fig. 4) for expert graders. The relevance map from the vehicle group revealed minor colour changes that appeared as small, irregular spots. However, in the group which tested the active formulation, visible colour changes are clearly more frequent, taking the shape of larger spots or patches, especially at the level of the forehead and at the level of the cheek, just below the eyes.

### Significance maps give a detailed insight on colour changes over time

**Figure 5:**
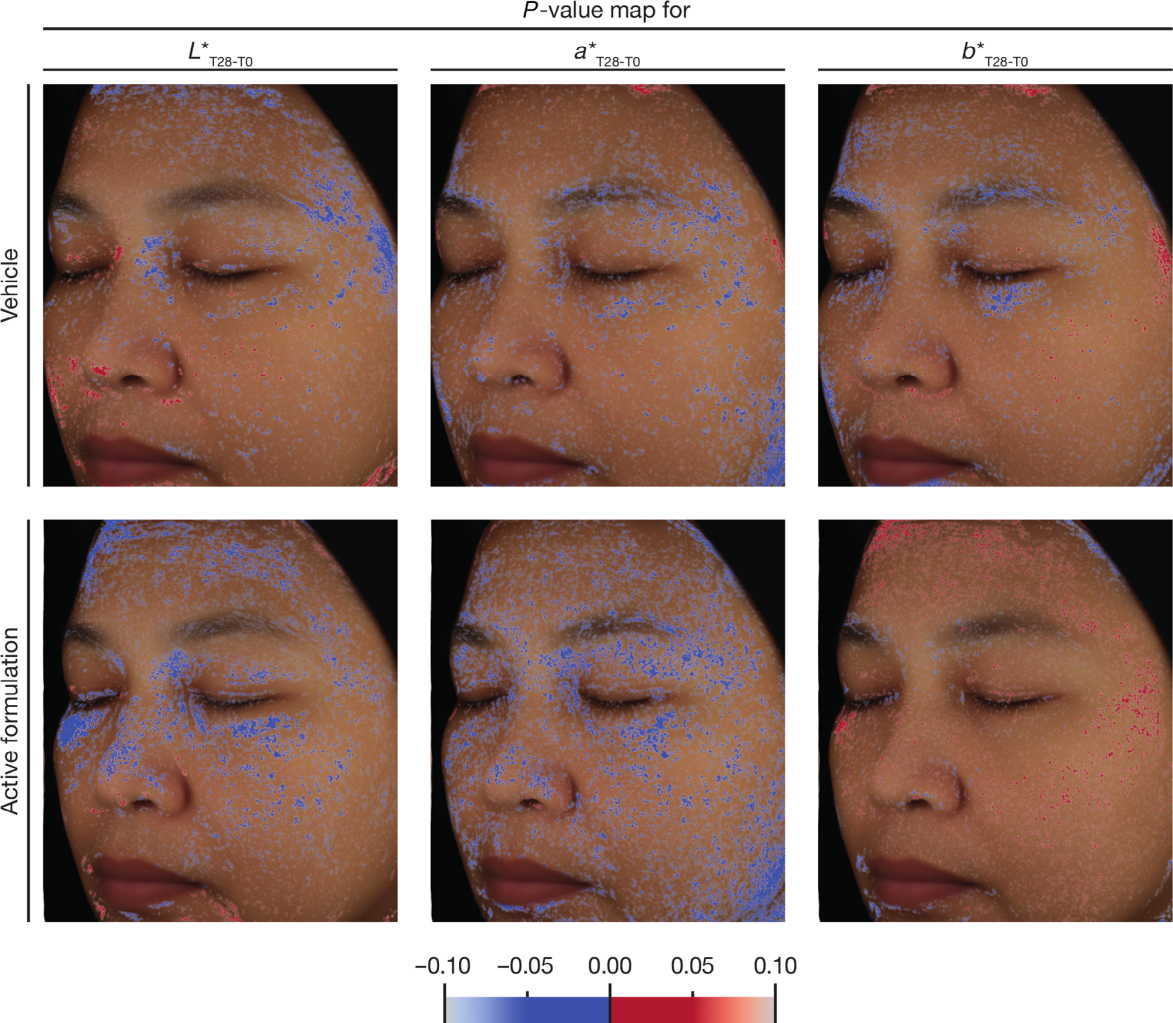
Significance maps (*P* < 0.05) for the changes in the L*, a* and b* parameters in the group which tested the vehicle emulsion and in the group which tested the active formulation.

To gain access to more data, we used the variations in each of the L*, a* and b* parameters to calculate the *P*‐value from a Student’s *t*‐test for each of these variations. This approach enabled us to generate, for every pixel of the reconstructed average facial side images, significance maps of L*a*b* changes over time. The results for such maps, onto which only data corresponding to significant changes in L*, a* or b* parameters (*P* < 0.05), are presented in Fig. 5.

In the case of subjects who applied the placebo, the significance map of the L* parameter shows little changes during the 28 days period of the test. On the contrary the significance map of the L* parameter for the group which tested the active formulation indicates a significant shift over higher L* values, over greater facial areas than for the vehicle‐treated group. This significantly lighter skin colour is particularly found around the eye, but also on the upper part of the cheek and the nose. It is measured, to a lesser extent, on the upper forehead.

As in the case of the significance map of the L* parameter, the significance map of the a* parameter shows little effect for the group which tested the vehicle for 28 days, but important changes in the case of the group which applied the active formulation. Indeed, this significance map shows large patches of significantly less red skin colour. This is particularly the case around the eyes, but also on the cheek, particularly the lower part and along the jawline, and, to a lower extent on the forehead.

Finally, in the case of the group which tested the vehicle, the significance map of the b* parameter shows almost no variations. In the case of the group which tested the active formulation, bigger patches of significantly increased b* values appeared on the forehead whereas no change was seen on the other parts of the face (Fig. 5 and Table 2), after 28 days of treatment.

## Discussion

Objective evaluation of skin colour changes provides crucial information on the condition of the skin and allows the effect of skin care treatments to be monitored. This can be especially suitable for early colour variation detection or study of colour contrast over the face. However, in this study, we only measured changes between baseline and after 28 days of treatment. Still, existing methods are poorly adapted to measuring colour changes, as well as the relevance and/or the significance of these changes over several subjects and over different time points. They are also unable to detect the exact localization of these changes among subjects and over extended periods of time. We used such a method to detect facial skin colour changes after a twice daily application of an anti‐ageing emulsion for 28 days. The vehicle group showed no significant colour evolution between the two time points. In contrast, the group which was treated with the active formulation showed decreased redness over the four predefined facial regions, and lighter skin at the level of the upper cheek.

These results demonstrate that it is possible to measure colour changes among several subjects and over time points. Nevertheless, such a classical method has limitations. Template matching allows approximate positioning of the predefined regions of interest over subjects’ faces but it requires manual adjustments that are time consuming and is subject to variations between subjects. In addition, colour data, and their changes over time, are the results of the average measurement made over the entire region of interest, therefore, leading to the risk of under‐estimating the effect depending on how large regions of interest are, and where the effect occurs. Finally, regions of interest might omit facial areas where an effect occurs, as it is our case for the decreased redness of the jawline.

To circumvent these limitations, we developed a new approach. A crucial feature of this approach is the quality of the reconstruction of the average face. This was achieved by automatic detection of 60 characteristic morphological points over both facial sides – the direct left facial side image and the mirror image of the right facial side – of all subjects and all time points. It is, then, the computer‐generated deformation of each characteristic morphological point to its average position that ensures the extremely high quality of the mean face reconstruction. It is thanks to this quality that we ensure correspondence of anatomical zones between subjects, or images, and time points. This is not only true for the main average face we reconstructed but also for all average facial side images we subsequently extracted from it, whatever treatment or time points. Therefore, it is this feature that enabled direct comparison of colour data from the very same morphological zones over the entire face and to compute colour changes occurring between time points.

Another important asset of this approach is the continuous mapping of results derived from colour changes over time onto the reconstructed facial images, using colour gradients. This feature provides immediate visibility of changes over the entire facial images. The first parameter we mapped using this approach is the relevance of colour change from the calculation of the Δ*E*‐values of every pixel, reporting onto the map, only data corresponding to visible colour changes (i.e. relevant colour changes). The relevance maps show no real effect in the group which tested the vehicle emulsion, but visible colour changes are obvious at the level of the forehead and just below the eyes, in the group which tested the active formulation. Still, relevance maps only provide information on the visibility and the location of colour changes. They give no information on the nature of the underlying colour changes and if these colour changes go in the expected direction. To do so, we analysed the L*, a* and b* parameters, and mapped significant changes, namely p‐values bellow 0.05 resulting from the Student’s t‐test performed on the changes in each of the L*, a* and b* parameters. Significance mapping proved to be a powerful tool to detect evolutions in each of these parameters. Not only did they show where colour changes occur, they also show the nature of the changes thanks to the two‐colour gradient used to map the results. Indeed, if no real significant evolution was observed in any of the L*a*b* parameters in the group which tested the vehicle emulsion, localization and significance of the evolution in the L*, a* and b* parameters are obvious in the significance maps. They also match the results obtained by the classical method we initially used but are more powerful as they do not rely on the *a priori* definition of regions of interest.

Interestingly, we found regional differences in whitening across the face, and additionally they do not necessarily match in both methods. Whitening differences across the face can arise due to differences in skin architecture, like thickness which would lead to different activity of the anti‐ageing product due to variable bioavailability. In addition, the visible perception of skin colour can vary due to variation in light refraction due to skin thickness variation. Differences in skin parameters like hydration or sebum content could also influence colour perception. Uneven skin tone, mottled pigmentation and age spots are all well known signs of ageing, and an anti‐ageing active may perform differently in each area. Furthermore, with regards to the two methods of significance and relevance mapping, we would like to point out that a change in skin colour can be significant but not relevant and vice versa. For example, a change in skin colour can be small with little variation over a large subject population making it significant but not relevant. However, the colour change could also be quite remarkable but with great variation and thus not be significant.

We chose to use a simple statistical approach due to the design of our study but more advanced, and suitable, tests could also be used to compare within subject variations (time) and between subject variations (placebo versus formulation) like generalized linear mixed models if the design of the study was different. It would be also interesting to add assumptions on the verification of the use of parametric tests, including normality and homoscedasticity tests. Not forgetting that tests are performed pixel by pixel, the relevance of using different tests onto two juxtaposed pixels will be discussed in an upcoming paper.

Taken together, both methods show that the formulation containing the anti‐ageing peptide lead to decreased redness and lighter skin, therefore, attenuating UV‐induced colour changes that accumulate with age 5, 6.

Large increases in skin yellowness have been associated with skin ageing 35, 36. Importantly, however, even the study of Bissett *et al*. with short‐term treatment with niacinamide, a typical anti‐ageing ingredient, showed a trend of increased yellowing of the skin compared with vehicle at the 4 week time point. In the longer term, the opposite occurred but the increases in yellowing were much more dramatic. As a result, the degree of yellowing may be important in the perception of ageing, health and attractiveness. In this respect in a recent investigation Malaysian Chinese subjects were asked to manipulate the skin colour of Chinese, Caucasian and African faces to make them look healthy and chose to increase skin yellowness to optimize this perception 37.

Moreover, some investigations have shown that increases in skin coloration from ingestion fruit and vegetables, some of which was associated with small increases in skin yellowness, were perceived positively for Caucasian and African subjects but not for Chinese subjects 38, 39, 40, 41, 42, 43. As a result, we do not consider the small increases in yellowing of the skin in our study as negative.

A general limitation of this study was the lack of comparison with clinical grading to determine how the colour difference at the pixel level can be compared with an overall visual difference. This will be the included in future studies, but the present study was focussed on method development using an anti‐ageing ingredient 27.

Still, the new image‐based continuous mapping approach we describe here is a much more powerful and a very accurate method. It enables, by a visual approach, to immediately identify and precisely delineate regions of the faces where colour changes occur, indicating not only the relevance of these colour changes but also the significance and the types of colour changes that occur over time and/or between groups. Therefore, our new approach helps to instantly identify areas of significant efficacy of skin care treatments and identifies regions of interest not readily visible for dedicated cosmetic/dermatological claims.

## Disclosure

P. Seroul is a former employee of Newtone Technologies, a company specialized in imaging solutions for life sciences. S. Gougeon and M. Cherel are full‐time employees of Newtone Technologies. R. Campiche and R. Voegeli are full‐time employees of DSM Nutritional Products Ltd., a global science‐based company working in the field of health, cosmetology, nutrition and materials and the manufacturer of the anti‐ageing peptide tested in the study. A.V. Rawlings is a consultant to DSM.

## Funding

This study was financially supported by DSM Nutritional Products Ltd., Basel, Switzerland.
